# The numb efficiency paradox: AI work pressure, affective numbing, and professional judgment in organizationally digitally mediated work

**DOI:** 10.3389/fpsyg.2026.1900940

**Published:** 2026-08-28

**Authors:** Shiyao Yin, Wenxuan Hu, Zirong Tian, Yifan Zhang

**Affiliations:** 1 Henan Provincial Center for Disease Control and Prevention, Zhengzhou, China; 2 Tax and Taxation Department, Belarusian State Economic University, Minsk, Belarus; 3 Media communication, Dankook University, Yongin-si, Gyeonggi-do, Republic of Korea; 4 College of Science and Technology Changchun, Changchun, China

**Keywords:** accounting employees, affective numbing, artificial intelligence, ethical vigilance, occupational resilience, professional skepticism, technostress

## Abstract

Artificial intelligence is reshaping accounting work by accelerating task cycles, expanding exception monitoring, and increasing reliance on automated recommendations. In accounting and auditing, these changes are consequential because work quality depends not only on efficient task completion, but also on ethical vigilance, professional skepticism, and careful evaluation of AI assisted outputs. This study introduces the numb efficiency paradox, a process in which accounting professionals may continue to meet work demands while becoming less affectively responsive to pressure, warning signs, and professional consequences. Using a single three wave longitudinal panel of accounting and auditing professionals recruited through occupation based prescreening and study specific eligibility screeners (T1, *n* = 512; T2, *n* = 417; T3, *n* = 361), the study examines AI related work pressure, occupational resilience, affective numbing, sustained work efficiency, ethical vigilance, and professional skepticism. The declining wave-specific sample sizes reflect panel attrition and quality screening rather than separate studies or newly recruited respondents. The results show that AI related work pressure was prospectively associated with both occupational resilience and affective numbing. Occupational resilience was prospectively associated with later sustained work efficiency. Affective numbing was not significantly associated with sustained work efficiency, but it was negatively associated with ethical vigilance and professional skepticism. AI autonomy and supervisor support showed beneficial direct associations with higher resilience and lower affective numbing, whereas the hypothesized interaction effects were not supported. These findings distinguish resilient efficiency from detached persistence and suggest that AI implementation should be evaluated not only by whether accounting work remains efficient, but also by whether professionals remain ethically vigilant and professionally skeptical when using AI supported systems.

## 1 Introduction

Accounting work has always required employees to manage deadlines, comply with rules, and detect errors with consequential professional and organizational implications. AI enabled systems are now changing the structure of these demands. They automate routine classifications, surface exceptions, accelerate close and reporting cycles, and require accountants and auditors to evaluate outputs generated by tools whose internal logic may not be fully transparent (Brown-Liburd et al., 2015; Kokina and Davenport, 2017). Although these systems can improve efficiency, they also intensify the expectation that professionals respond quickly while remaining accountable for judgment quality (Issa et al., 2016). This tension is especially important in accounting and auditing because work quality cannot be reduced to task completion. It depends on ethical vigilance, professional skepticism, resistance to automation bias, and careful questioning of outputs that appear efficient but remain professionally uncertain (Hurtt, 2010; Nelson, 2009). In this context, an employee who continues to meet productivity targets while becoming less responsive to anomalies, warnings, or ethical discomfort represents a form of professional quality risk that conventional efficiency metrics are unlikely to detect (Mautz and Sharaf, 1961).

Existing research on AI enabled work and technostress has often treated performance and strain as separate outcomes, with organizations assumed to balance productivity gains against employee well-being costs (Brougham and Haar, 2018; Tarafdar et al., 2019). Related work on occupational stress similarly emphasizes how job demands generate strain, adaptation, or resource loss over time (Salanova et al., 2013). This framing overlooks a more subtle possibility: efficiency may remain visible while the psychological process sustaining it shifts from engaged adaptation to affective detachment. The present study develops the concept of the numb efficiency paradox to capture this possibility. The paradox does not refer to a logical contradiction, but to an evaluative trap in which the dominant indicator of AI implementation, task efficiency, fails to signal a deterioration that is psychologically meaningful and professionally consequential. Employees exposed to repeated AI related pressure may continue to satisfy quantitative performance expectations while becoming less sensitive to warning cues, ethical discomfort, and professional doubt. The paradox is therefore structural rather than merely individual. When organizations evaluate AI enabled accounting work primarily through speed, output volume, or completion rates, they may overlook affective numbing, a psychological mechanism that can quietly weaken the professional sensitivity on which judgment quality depends.

**Figure 1:**
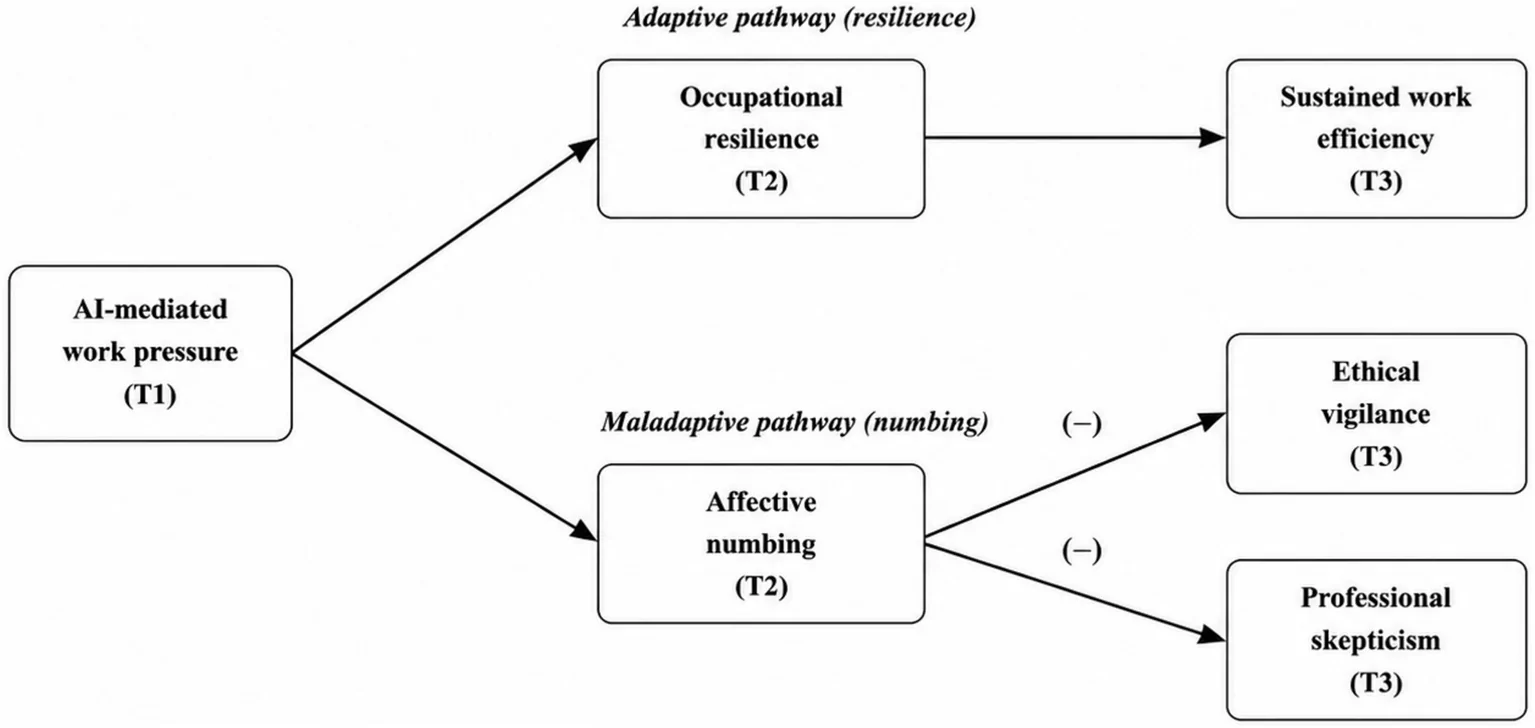
Conceptual framework of the dual adaptation pathways underlying the numb efficiency paradox.

This study contributes to the literature by introducing affective numbing as a distinct maladaptive adaptation pathway under AI-mediated work pressure, grounded in conservation of resources theory and affective processing perspectives on repeated stressor exposure (Hobfoll, 1989). As illustrated in Figure 1, the proposed framework distinguishes two parallel adaptation pathways through which AI-mediated work pressure may shape subsequent professional outcomes: an adaptive pathway characterized by occupational resilience and a maladaptive pathway characterized by affective numbing. This concept describes a condition in which employees may remain behaviorally functional while becoming less affectively responsive to pressure, anomalies, and professional consequences. The study also distinguishes occupational resilience from affectively numbed persistence. Both may allow employees to maintain work output, but they reflect different psychological processes and have different implications for later work quality. Resilience reflects adaptive recovery and continued professional engagement, whereas numbing reflects detached persistence that may be associated with lower ethical vigilance and professional skepticism. By linking AI-mediated pressure to professional judgment outcomes in accounting, the study shifts attention from whether AI-assisted work remains productive to whether the psychological conditions necessary for responsible accounting judgment are being preserved.

This perspective also reflects the broader transformation of accounting and auditing under AI-enabled technologies. Recent studies have shown that AI systems are reshaping audit procedures, accounting workflows, and professional responsibilities while expanding the role of data-driven decision support in practice (Appelbaum et al., 2017; Cao et al., 2015; Greenman, 2017; Sun and Vasarhelyi, 2017). At the same time, digitalization has introduced new challenges for professional judgment, requiring accountants and auditors to balance technological efficiency with ethical accountability, professional skepticism, and evolving regulatory expectations (Raphael, 2017; Moll and Yigitbasioglu, 2019; International Auditing and Assurance Standards Board, 2023; Munoko et al., 2020; Payne and Ramsay, 2005). Although occupational research has documented how prolonged work demands may contribute to burnout, disengagement, and changes in work engagement (Kristensen et al., 2005; Demerouti et al., 2010), considerably less attention has been paid to the possibility that employees may continue to perform efficiently while becoming affectively detached from the professional cues necessary for responsible judgment. Addressing this gap provides the conceptual foundation for the present investigation of the numb efficiency paradox.

## 2 Theory and hypotheses

This section develops the theoretical logic for the numb efficiency paradox. It first defines AI mediated work pressure in accounting, then distinguishes occupational resilience from affective numbing, and finally explains how AI autonomy and supervisor support may relate to these adaptation pathways.

### 2.1 AI-mediated work pressure in accounting

AI mediated work pressure refers to the experienced intensification of work demands created by AI supported systems. In accounting, such pressure includes the accelerated pace of AI enabled workflows, continuous adaptation to changing tools, monitoring expectations, output verification demands, and responsibility for AI assisted decisions. Unlike general workload, AI work pressure combines cognitive, temporal, and evaluative demands because employees are not only asked to complete more tasks, but also to coordinate professional judgment with tools that appear fast and precise while still requiring careful verification (Dowling and Leech, 2014; Kokina and Davenport, 2017). This pressure may be associated with two forms of persistence. One form is adaptive, as employees may learn to prioritize, recover, and integrate AI tools into professional routines, which is consistent with appraisal and coping perspectives on work stress (Cavanaugh et al., 2000; Lazarus and Folkman, 1984). Another form is maladaptive, as repeated pressure, ambiguity, and responsibility for AI assisted outputs may encourage emotional distancing and reduced affective responsiveness to professional warning signs. Resilience and numbing should therefore not be treated as simple opposites. They may coexist because an employee can continue adapting in some aspects of work while becoming less emotionally responsive in others. From a conservation of resources perspective, AI work pressure threatens cognitive, affective, and professional resources at the same time (Hobfoll, 1989). Employees who perceive sufficient resource reserves, or who receive organizational resources such as autonomy and supervisor support, are more likely to appraise AI pressure as a challenge and invest effort in adaptive coping, resulting in occupational resilience. Employees who experience sustained resource loss without adequate replenishment, by contrast, may conserve remaining resources by reducing emotional investment, which provides the psychological basis for affective numbing. These pathways are not mutually exclusive. An employee may show resilient task adaptation in some domains while conserving emotional resources through numbing in others, producing the visible pattern of maintained efficiency combined with weakened professional sensitivity that motivates this study.

*H1:* AI-mediated work pressure is positively associated with occupational resilience.

*H2:* AI-mediated work pressure is positively associated with affective numbing.

### 2.2 Occupational resilience and affective numbing as dual adaptation pathways

Resilience involves active adaptation, recovery, prioritization, and continued professional engagement despite strain (Bandura, 1997; Bonanno, 2004). A resilient accounting employee remains capable of learning, regulating pressure, preserving professional purpose, and sustaining attention to judgment quality. From a conservation of resources perspective, resilience reflects the capacity to protect, restore, and reinvest resources when work demands become intense (Hobfoll, 1989). In AI assisted accounting work, this capacity is especially important because employees must not only maintain output, but also continue questioning automated suggestions, monitoring exception signals, and evaluating whether AI generated outputs remain professionally defensible. Occupational resilience should therefore be associated with sustained work efficiency, because resilient employees are better able to recover from pressure, organize competing demands, and preserve work continuity. It should also be relevant to professional engagement outcomes such as ethical vigilance and professional skepticism, because judgment quality depends on continued attention, doubt, and responsiveness to evidence rather than on task completion alone.

Affective numbing represents a different form of persistence. It involves reduced emotional responsiveness, flattened concern, and a functional but detached mode of continued work. An affectively numbed employee may still complete tasks and meet deadlines, but may react less strongly to pressure, exception alerts, error warnings, ethical discomfort, or professional consequences. This construct is related to but distinct from adjacent concepts. Burnout emphasizes exhaustion and depersonalization, whereas affective numbing emphasizes blunted responsiveness to recurring pressure and professional warning cues while task engagement may continue (Maslach et al., 2001). Emotional exhaustion refers primarily to depletion, while numbing refers to detachment and reduced affective sensitivity. Disengagement implies withdrawal from work, whereas numbing can coexist with continued task completion. Emotional labor research shows that workers can regulate outward expression while internal experience shifts, but affective numbing concerns reduced responsiveness rather than role based display rules (Ashforth and Humphrey, 1993). Moral disengagement involves cognitive justification of questionable behavior after or during action, whereas affective numbing operates at the level of sensitivity, reducing the discomfort that would ordinarily trigger moral attention (Bandura, 1999). Automation bias concerns overreliance on automated outputs, while affective numbing concerns the worker’s emotional and evaluative responsiveness under AI mediated pressure (Parasuraman and Riley, 1997). These distinctions are not merely taxonomic, because they imply different measurement expectations. Burnout and emotional exhaustion scales typically assess depletion and withdrawal motivation, while affective numbing items should capture maintained task functioning alongside reduced affective responsiveness. This distinction matters in accounting because professional judgment often depends on discomfort, doubt, and sensitivity to anomalies. Professional skepticism has been described as a questioning and evidence sensitive orientation in audit judgment, and it can be shaped by fraud risk assessment and client related pressures (Hurtt, 2010; Nelson, 2009). Affective numbing may allow employees to continue task completion in the short term, but its association with sustained efficiency is theoretically uncertain and may be weaker than the resilience pathway. Its clearer professional risk is that it may reduce sensitivity to warning signs, ethical discomfort, ambiguous anomalies, questionable estimates, and overreliance on AI outputs.

*H3:* Occupational resilience is positively associated with sustained work efficiency.

*RQ1:* Is affective numbing associated with sustained work efficiency after accounting for occupational resilience and controls?

*H4a:* Affective numbing is negatively associated with ethical vigilance.

*H4b:* Affective numbing is negatively associated with professional skepticism.

### 2.3 AI autonomy, supervisor support, and adaptation under AI work pressure

The following conceptual summary table clarifies how affective numbing differs from adjacent constructs used in the manuscript. Table 1 is intended to show that affective numbing is not proposed as another label for burnout, automation bias, moral disengagement, or emotional exhaustion, but as a narrower state of reduced affective responsiveness under continued task functioning.

**Table 1:** Conceptual distinctions between affective numbing and related psychological constructs.

Construct	Primary focus	Relation to affective numbing	Key distinction
Emotional exhaustion	Resource depletion and fatigue	Conceptually adjacent	Numbing emphasizes flattened responsiveness rather than felt depletion.
Burnout/depersonalization	Chronic exhaustion and distancing from work or clients	Adjacent but broader	Numbing can coexist with continued task completion and does not require withdrawal.
Automation bias	Overreliance on automated outputs	Distinct AI judgment risk	Numbing concerns affective sensitivity under pressure, not only acceptance of AI output.
Moral disengagement	Cognitive justification of questionable behavior	Downstream or adjacent risk	Numbing reduces warning sensitivity before explicit justification may occur.
Occupational resilience	Adaptive recovery and continued professional engagement	Contrasting pathway	Resilience preserves engaged functioning; numbing reflects detached persistence.

AI autonomy refers to employees’ perceived discretion over when, how, and whether AI outputs are incorporated into accounting judgment. When autonomy is higher, employees can treat AI as a resource rather than a controlling system. Supervisor support can also protect professional judgment by legitimizing concerns, encouraging questions, and valuing judgment beyond automated efficiency metrics. These contextual resources may shape whether AI pressure is associated with resilient adaptation or emotional shutdown. The expected buffering effects should be interpreted cautiously, however, because organizational resources may also operate as general resources with direct associations rather than as moderators of pressure slopes.

*H5a:* AI autonomy is negatively associated with affective numbing, after controlling for AI work pressure and supervisor support.

*H5b:* Supervisor support is negatively associated with affective numbing, after controlling for AI work pressure and AI autonomy.

*H5c:* AI autonomy and supervisor support each moderate the positive association between AI work pressure and affective numbing, such that the association is weaker when autonomy or support is higher. This moderation prediction is examined as a secondary test given theoretical uncertainty about whether organizational resources reduce numbing differentially across pressure levels or primarily operate as main-effect buffers.

## 3 Materials and methods

This section describes the longitudinal panel design, participant eligibility and matching procedures, measurement development, construct validation, and statistical approach used to test the proposed model.

### 3.1 Participants and procedure

The study used a single three wave longitudinal survey design rather than three separate studies. Accounting and auditing professionals were recruited at T1 through Prolific Academic using occupation based prescreening filters and study specific eligibility screeners. Sampling was restricted to participants in the United States, the United Kingdom, Canada, and Australia, where AI assisted accounting tools such as automated anomaly detection, AI supported close processes, and machine learning based risk flagging are increasingly used in professional practice, and where accounting qualifications are formally regulated by professional bodies. Eligibility required active employment in a paid accounting or auditing role, including financial accounting, audit, tax, management accounting, or shared services; at least weekly use of AI enabled or automation assisted work tools as part of regular job duties; and possession of a recognized accounting qualification or current enrollment in a professional accounting program, such as CPA, ACA, ACCA, or CMA. AI enabled or automation assisted tools were defined as systems that automatically classify transactions, surface exceptions, generate predictive estimates, or produce recommendations requiring professional review. Eligibility was checked through Prolific prescreening information, self reported occupational information, professional qualification status, AI use frequency, and consistency checks embedded in the survey. Participants who failed one or more eligibility screeners were excluded before the final T1 sample was established. To reduce satisficing and inattentive responding, two attention check items were included in the T1 survey. Participants who failed either attention check were excluded from all analyses, resulting in the exclusion of 31 respondents before the final T1 total of 512 was reached. Data were collected online through individual survey links, and participants were not identifiable to their employers. Participants were followed across waves using Prolific participant IDs and study-specific matching fields; no new participants were added at T2 or T3. Noncompleters at T2 or T3 received up to two reminder messages before being classified as attrited.

At T1, participants reported AI mediated work pressure, AI autonomy, supervisor support, baseline well being, and demographic controls. At T2, approximately six weeks later, participants reported occupational resilience and affective numbing. At T3, approximately twelve weeks after baseline, participants reported sustained work efficiency, ethical vigilance, and professional skepticism. The six week interval was selected to balance theoretical and practical considerations: it is long enough for repeated exposure to AI mediated pressure to be associated with adaptation processes, while short enough to reduce recall error and excessive panel loss in an online professional sample. This temporal separation was designed to reduce same time measurement bias and to distinguish pressure exposure, psychological adaptation, and later work outcomes; however, it does not establish causality. The same consent, confidentiality, and voluntary participation procedures were used at all waves. Participants were informed at T1 that the project involved follow up surveys, and each follow up invitation reiterated that participation was voluntary and that they could withdraw without penalty. The models included age, gender, tenure, weekly work hours, and baseline well being as controls because these variables may shape responses to work pressure and later functioning. Missingness was handled through available case estimation for each model, and attrition was examined descriptively before the focal analyses. The final analytic sample comprised 361 participants who completed all three waves. Compared with participants who completed only T1, retained participants reported modestly lower AI work pressure (d = −0.18, *p* = 0.067), higher supervisor support (d = 0.24, *p* = 0.016), and higher baseline well being (d = 0.23, *p* = 0.013). This pattern suggests that the estimates may conservatively represent the experiences of employees exposed to more intense AI related pressure and may underestimate associations among the most affected professionals.

### 3.2 Measures

All focal variables were measured on five point Likert type scales ranging from 1 = strongly disagree to 5 = strongly agree. Scale development followed a deductive approach, with construct definitions guiding item selection, adaptation, and refinement (Hinkin, 1998; DeVellis, 2016). Items for established constructs, including occupational resilience, supervisor support, and baseline well being, were adapted from validated scales and revised to fit AI assisted accounting contexts. Three accounting practitioners and two organizational psychologists reviewed the wording for content validity, contextual appropriateness, and conceptual clarity. Affective numbing was operationalized as a newly specified construct. Item development followed the procedure described by DeVellis (2016), including item generation from the conceptual definition, expert review, and refinement based on interrater agreement on content representativeness. All retained items achieved a content validity index above 0.80. AI mediated work pressure and AI autonomy were developed through iterative item generation and expert review using the same logic. AI mediated work pressure captured the speed, monitoring, learning, and evaluative pressure created by AI supported accounting systems. AI autonomy captured perceived discretion over when and how AI outputs were incorporated into professional work. Affective numbing captured emotional flattening and detached persistence under pressure. Occupational resilience captured recovery, flexible adaptation, and preserved professional purpose. Sustained efficiency captured task completion, deadline reliability, and steady work pace. Ethical vigilance and professional skepticism captured attention to ethical risk, willingness to question AI output, and evidence based review of automated suggestions. Full item wording and exploratory factor evidence are reported in Appendix A. Because affective numbing is a newly specified construct and the focal validation evidence comes from the same panel used for hypothesis testing, the scale should be interpreted as an emerging measure rather than as an established psychometric instrument.

Additional analyses were conducted to assess whether affective numbing was empirically distinguishable from its nearest conceptual neighbors. In item screening and exploratory factor assessment, affective numbing items clustered around reduced affective responsiveness and detached persistence rather than around general exhaustion, depersonalization, or automation reliance. Confirmatory tests then compared affective numbing with emotional exhaustion using the three item exhaustion subscale of the Maslach Burnout Inventory General Survey (Maslach et al., 2001). A two factor confirmatory model separating affective numbing and emotional exhaustion fit substantially better than a merged one factor model, with ΔCFI = 0.041 and ΔRMSEA = −0.013, and the HTMT ratio was 0.71, below the 0.85 threshold. Affective numbing was also compared with depersonalization from the MBI GS. The two factor model again fit better than the merged model, with ΔCFI = 0.038, and the HTMT ratio was 0.68. A further comparison used a three item automation bias index adapted from Parasuraman and Riley (1997), which assessed uncritical acceptance of automated outputs. The HTMT ratio between affective numbing and automation bias was 0.44, indicating limited conceptual overlap. These results support the interpretation that affective numbing captures maintained task engagement combined with reduced affective responsiveness, rather than general burnout, depersonalization, or automation reliance. Because these comparisons were conducted within the same sample as the focal analyses, they should be treated as preliminary evidence of construct distinctiveness rather than definitive validation. Replication with independent samples, split-sample EFA-CFA designs, behavioral indicators, and multi method measurement remains necessary. To support longitudinal comparisons, measurement invariance was evaluated through configural, metric, and scalar models, and the results supported at least partial scalar invariance for all constructs used in cross wave analyses (Vandenberg and Lance, 2000).

Exploratory factor analyses were also conducted on the item-level data using principal-axis factoring. For each focal construct, the first eigenvalue exceeded 1.00 and the second eigenvalue was below 1.00, supporting the intended one-factor structure at the construct level. Across the construct-specific extractions, factor loadings ranged from 0.519 to 0.744, and the overall nine-factor item-level extraction showed that all items loaded primarily on their expected theoretical factor. Detailed eigenvalues, factor loadings, and extraction communalities are reported in Appendix A.

### 3.3 Data analysis

All analyses were conducted in R version 4.3.1. Structural equation modeling was performed using lavaan (Rosseel, 2012), with supporting procedures conducted through semTools (Jorgensen et al., 2022). Robustness checks used HC3 standard errors estimated with the sandwich package. The analysis began with sample characteristics and attrition patterns, followed by reliability, composite reliability, average variance extracted, correlations, HTMT ratios, exploratory item screening, and measurement model comparisons. Common method bias was evaluated through a single factor comparison and a common latent factor model. Longitudinal path models were then estimated to test whether T1 AI mediated work pressure was prospectively associated with T2 occupational resilience and affective numbing, and whether T2 adaptation processes were prospectively associated with T3 sustained work efficiency, ethical vigilance, and professional skepticism after controlling for baseline well being and demographic covariates. Indirect associations were evaluated using Monte Carlo confidence intervals. Robustness checks examined whether the focal estimates remained stable after including role fixed effects, restricting the sample to high AI use participants, and testing alternative professional quality outcomes. Longitudinal measurement invariance was assessed through configural, metric, and scalar models, with chi square difference tests and change in CFI less than 0.010 used to evaluate successive constraints (Vandenberg and Lance, 2000).

## 4 Results

This section reports the empirical evidence in four steps: sample retention and measurement quality, longitudinal structural associations, indirect associations and robustness checks, and explained variance for the endogenous outcomes.

### 4.1 Measurement model and descriptive evidence

The T1 sample included 512 accounting and auditing professionals recruited through the professional panel procedures described in Section 3.1. Retention was 81.4% at T2 and 70.5% at T3. Attrition analyses indicated modest selectivity. Participants with higher AI mediated work pressure were somewhat less likely to remain through T3, while retained participants reported higher supervisor support and baseline well being. This pattern suggests that the retained sample may underrepresent employees experiencing the most intense AI related pressure. The observed attrition pattern should therefore be considered when interpreting the longitudinal estimates, and it is revisited as a limitation in Section 5.3.

Reliability and validity evidence supported the adequacy of the measurement model. Reliability values ranged from acceptable to strong, with Cronbach’s alpha ranging from 0.743 to 0.827 and composite reliability ranging from 0.839 to 0.874, consistent with conventional reporting thresholds (Hair et al., 2019). Average variance extracted values met or exceeded 0.50 for eight of the nine constructs. The only exception was AI mediated work pressure, with AVE = 0.478, which was retained because its composite reliability was acceptable at CR = 0.846 and because the construct was theoretically broad, spanning cognitive, temporal, and evaluative demand dimensions. All HTMT ratios were below 0.85, with values ranging from 0.24 to 0.79, supporting discriminant validity among the focal constructs (Henseler et al., 2015). The nine factor measurement model showed acceptable fit, χ^2^(866) = 1432.84, CFI = 0.939, TLI = 0.928, RMSEA = 0.045 [0.041, 0.049], SRMR = 0.052, and fit better than the nested alternatives. The single factor model fit poorly, with CFI = 0.781 and RMSEA = 0.088. The common latent factor model improved fit only marginally, with ΔCFI = 0.007, and did not substantially alter the focal path estimates, as the maximum absolute change in standardized path coefficients was below 0.03. These results suggest that common method variance remains possible but is unlikely to account for the substantive findings. Longitudinal measurement invariance tests supported configural and metric invariance for all constructs and at least partial scalar invariance, allowing meaningful cross wave comparisons.

### 4.2 Lagged path results

The longitudinal path estimates generally supported the core pattern of the numb efficiency paradox, although not all expected relationships were observed. AI work pressure was positively associated with both occupational resilience and affective numbing, indicating that AI related demands may activate adaptive coping and detached persistence at the same time. Occupational resilience was positively associated with later sustained work efficiency, whereas affective numbing was not significantly associated with sustained efficiency. This null association suggests that affectively detached persistence should not be interpreted as a reliable source of later efficiency in the present models. Affective numbing was, however, negatively associated with ethical vigilance and professional skepticism, indicating that its more consistent consequence lies in reduced professional sensitivity rather than reduced task output. AI autonomy and supervisor support showed beneficial direct associations with higher resilience and lower numbing, but the hypothesized interaction effects were not supported. Overall, the evidence supported H1, H2, H3, H4a, and H4b, while RQ1 did not indicate a reliable efficiency association for affective numbing and the secondary moderation tests were not supported. Explained variance values are summarized in Table 2. The models explained moderate variance in the T2 adaptation outcomes, with R^2^ = 0.28 for occupational resilience and R^2^ = 0.34 for affective numbing. The T3 outcome equations showed lower explained variance, with R^2^ = 0.18 for sustained work efficiency, R^2^ = 0.21 for ethical vigilance, and R^2^ = 0.19 for professional skepticism. This pattern is consistent with the temporal distance between T2 adaptation processes and T3 outcomes, as well as the modest attrition related restriction of range noted earlier.

**Table 2:** Sample characteristics and retention.

Characteristic	Category	n or M	% or SD
Initial sample	T1 completed	512	100.0
Retention	T2 completed	417	81.4
Retention	T3 completed	361	70.5
Age	Years	35.57	7.45
Tenure	Years	7.71	3.75
Weekly work hours	Hours	47.98	6.98
Gender	Female	282	55.1
Male	230	44.9	
Accounting role	Audit	120	23.4
Financial accounting	166	32.4	
Management accounting	74	14.5	
Shared service	75	14.6	
Tax	77	15.0	
AI use frequency	Daily	214	41.8
Several times weekly	237	46.3	
Weekly	61	11.9	
Education	Associate degree	83	16.2
Bachelor degree	358	69.9	
Master degree or above	71	13.9	

Percentages are based on the T1 sample.

Table 2 summarizes sample characteristics across the three waves. The T1 sample comprised 512 accounting and auditing professionals (55.1% female; M age = 35.57, SD = 7.45; M tenure = 7.71 years, SD = 3.75). The largest role group was financial accounting (32.4%), followed by audit (23.4%), tax (15.0%), shared service (14.6%), and management accounting (14.5%). AI tool use was intensive: 41.8% reported daily use and 46.3% several times per week, with only 11.9% reporting weekly use. Most participants held a bachelor degree (69.9%). Retention was 81.4% at T2 (*n* = 417) and 70.5% at T3 (*n* = 361), indicating moderate but acceptable attrition over the twelve-week study period.

Table 3 presents attrition analyses comparing T1-only completers with participants retained through T3. Retained participants reported modestly lower AI-mediated work pressure (M = 2.98 vs. 3.07, d = −0.18, *p* = 0.067) and meaningfully higher supervisor support (M = 3.04 vs. 2.89, d = 0.24, *p* = 0.016) and baseline well-being (M = 3.04 vs. 2.90, d = 0.23, *p* = 0.013). AI autonomy showed no significant difference (d = 0.13, *p* = 0.198). Effect sizes were small across all variables (|d| no more than 0.24), indicating that attrition selectivity was modest. These patterns suggest that estimates derived from the retained sample may conservatively underrepresent associations among the most pressured employees.

**Table 3:** Attrition analysis.

Variable	Retained M	Dropped M	Difference	Cohen d	*p*
AI-mediated work pressure	2.980	3.073	−0.093	−0.177	0.067
AI autonomy	3.025	2.952	0.073	0.125	0.198
Supervisor support	3.040	2.894	0.146	0.235	0.016
Baseline well-being	3.036	2.901	0.135	0.227	0.013

Positive differences indicate higher retained-participant means.

Table 4 reports reliability and convergent validity statistics for all nine constructs. Cronbach’s alpha ranged from 0.743 (AI autonomy) to 0.827 (affective numbing) and composite reliability (CR) ranged from 0.839 to 0.874, all meeting conventional thresholds. AVE values ranged from 0.478 to 0.632; eight of nine constructs exceeded the 0.50 benchmark. The exception, AI-mediated work pressure (AVE = 0.478), was retained because its CR (0.846) substantially exceeded the minimum and its HTMT ratios with all other constructs remained below 0.85. Affective numbing demonstrated the strongest reliability (alpha = 0.827, CR = 0.874, AVE = 0.537), supporting its psychometric adequacy as a newly developed construct. Construct means clustered near the scale midpoint (range: 2.988–3.016), with standard deviations between 0.527 and 0.641.

**Table 4:** Reliability, CR, and AVE.

Construct	Items	M	SD	Alpha	CR	AVE	Decision
AI-mediated work pressure	6	3.007	0.527	0.781	0.846	0.478	Retained with caution
AI autonomy	4	3.003	0.585	0.743	0.839	0.565	Passed
Supervisor support	4	2.997	0.625	0.806	0.873	0.632	Passed
Baseline well-being	4	2.996	0.599	0.769	0.853	0.591	Passed
Occupational resilience	5	3.016	0.614	0.810	0.868	0.569	Passed
Affective numbing	6	3.007	0.591	0.827	0.874	0.537	Passed
Sustained work efficiency	4	2.997	0.641	0.764	0.850	0.586	Passed
Ethical vigilance	5	2.997	0.613	0.805	0.865	0.562	Passed
Professional skepticism	4	2.988	0.624	0.759	0.848	0.583	Passed

Alpha = Cronbach’s alpha; CR = composite reliability; AVE = average variance extracted. AI mediated work pressure was retained with caution because its AVE was slightly below 0.50, while CR was acceptable and discriminant validity was supported by HTMT evidence.

Table 5 reports bivariate correlations among the focal variables. AI mediated work pressure showed its strongest positive correlation with affective numbing (r = 0.483), providing preliminary support for the pressure to numbing pathway. Its bivariate correlation with occupational resilience was close to zero (r = 0.018), indicating that the pressure to resilience relationship should be interpreted through the adjusted longitudinal model rather than through the zero order association alone. This pattern is plausible because AI pressure was negatively correlated with AI autonomy and supervisor support, whereas those contextual resources were positively associated with resilience. Affective numbing was negatively correlated with ethical vigilance (r = −0.287) and professional skepticism (r = −0.318), consistent with the expectation that numbing is more closely related to reduced professional sensitivity than to reduced task output. Sustained work efficiency showed weak correlations with ethical vigilance (r = 0.049) and professional skepticism (r = 0.066), supporting the view that efficiency and professional judgment sensitivity are empirically distinct outcomes.

**Table 5:** Descriptive correlations.

No	Variable	AIP	AIA	SUP	BWB	RES	NUMB	EFF	ETHV	SKEP
1	AI mediated work pressure	1.000								
2	AI autonomy	−0.147	1.000							
3	Supervisor support	−0.124	0.113	1.000						
4	Baseline well being	−0.219	0.119	0.260	1.000					
5	Occupational resilience	0.018	0.299	0.461	0.337	1.000				
6	Affective numbing	0.483	−0.276	−0.257	−0.376	−0.166	1.000			
7	Sustained work efficiency	0.260	0.117	0.068	0.040	0.315	0.164	1.000		
8	Ethical vigilance	−0.190	0.077	0.362	0.190	0.323	−0.287	0.049	1.000	
9	Professional skepticism	−0.209	0.305	0.230	0.221	0.348	−0.318	0.066	0.154	1.000

AIP = AI mediated work pressure; AIA = AI autonomy; SUP = supervisor support; BWB = baseline well being; RES = occupational resilience; NUMB = affective numbing; EFF = sustained work efficiency; ETHV = ethical vigilance; SKEP = professional skepticism. Correlations are computed from available cases.

**Figure 2:**
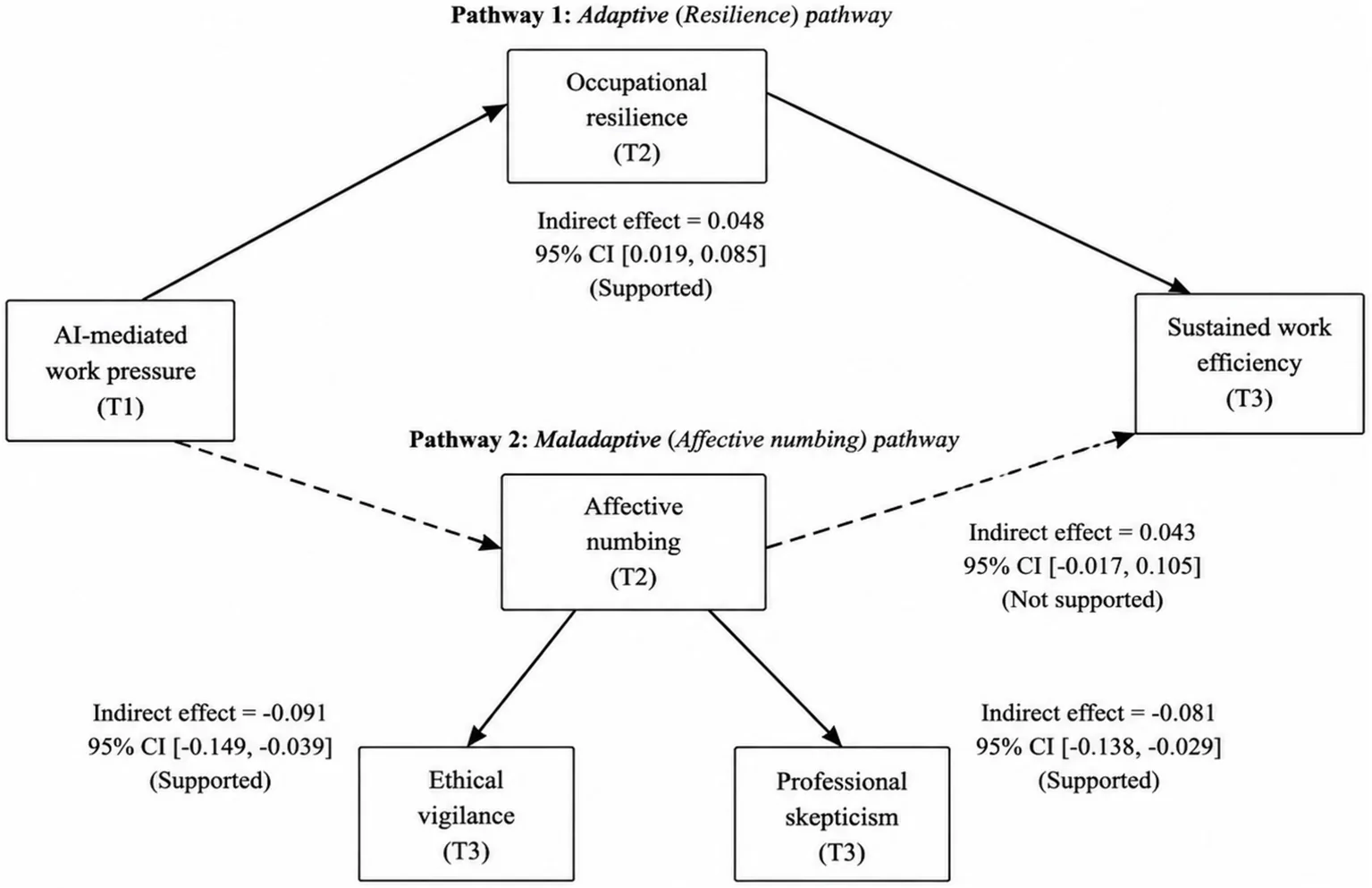
Dual adaptation pathways underlying the numb efficiency paradox: longitudinal indirect effects of AI-mediated work pressure.

Table 6 and Figure 2 present fit statistics for the focal measurement model and four comparison models. The nine factor measurement model showed acceptable fit: χ^2^(866) = 1432.84, CFI = 0.939, TLI = 0.928, RMSEA = 0.045 [90% CI: 0.041, 0.049], SRMR = 0.052. Merging occupational resilience and affective numbing into a single factor worsened model fit, with ΔCFI = −0.037 and ΔRMSEA = 0.011, supporting their empirical separability. Merging sustained work efficiency, ethical vigilance, and professional skepticism also worsened fit, with ΔCFI = −0.045, indicating that these T3 outcomes should be treated as distinct constructs. The single factor model fit poorly, with CFI = 0.781 and RMSEA = 0.088, suggesting that a single common factor cannot explain the observed covariance structure. The common latent factor model improved fit only marginally, with ΔCFI = 0.007, and substantive path estimates changed by less than 0.03 in absolute value. These results do not eliminate the possibility of common method variance, but they suggest that it is unlikely to account for the focal conclusions.

**Table 6:** Measurement model and common-method checks.

Model	Chi-square	df	CFI	TLI	RMSEA	SRMR	Interpretation
Nine factor measurement model	1432.840	866	0.939	0.928	0.045	0.052	Acceptable focal model
Merged resilience and numbing model	1685.260	874	0.902	0.889	0.056	0.064	Inferior Fit
Merged efficiency, vigilance, skepticism model	1744.910	875	0.894	0.881	0.059	0.067	Inferior Fit
Single-factor model	2318.480	902	0.781	0.758	0.088	0.094	Poor fit
Common latent factor model	1378.330	857	0.946	0.934	0.043	0.050	Limited CMV concern

Model comparisons evaluate whether the focal factor structure fits better than plausible alternative specifications. CMV = common method variance.

Table 7 presents the full set of longitudinal path estimates. AI work pressure was prospectively associated with both occupational resilience (β = 0.149, SE = 0.042, *p* < 0.001) and affective numbing (β = 0.391, SE = 0.047, *p* < 0.001), supporting H1 and H2. The positive pressure-to-resilience association (β = 0.149) coexists with the near-zero bivariate correlation reported in Table 3 (r = 0.018) because the longitudinal model controls for supervisor support (β = 0.403) and AI autonomy (β = 0.242), both of which are simultaneously positive predictors of resilience and negatively correlated with AI pressure. Partialling out these covariates reveals the unique positive longitudinal association between AI pressure and resilience that is suppressed in the bivariate association. The pressure-to-numbing association (β = 0.391) was more than twice the magnitude of the pressure-to-resilience association (β = 0.149), indicating that sustained AI pressure was more strongly associated with maladaptive detachment than adaptive recovery. Occupational resilience was prospectively associated with sustained work efficiency (β = 0.324, *p* < 0.001; H3 supported), ethical vigilance (β = 0.211, *p* = 0.001), and professional skepticism (β = 0.257, *p* < 0.001). Affective numbing was not significantly associated with efficiency (β = 0.108, *p* = 0.158; RQ1 null), but it was significantly associated with lower ethical vigilance (β = −0.234, *p* < 0.001; H4a) and lower professional skepticism (β = −0.208, *p* = 0.002; H4b). All four interaction terms were non-significant (all |beta| no more than 0.024, all *p* > 0.50), indicating no moderation.

**Table 7:** Longitudinal path estimates.

Outcome	Predictor	β	SE	95% CI	*p*
Occupational resilience	AI work pressure	0.149	0.042	[0.067, 0.232]	*p* < 0.001
	AI autonomy	0.242	0.035	[0.172, 0.311]	*p* < 0.001
	Supervisor support	0.403	0.043	[0.319, 0.487]	*p* < 0.001
	AI pressure x AI autonomy	−0.024	0.038	[−0.098, 0.050]	*p* = 0.525
	AI pressure x supervisor support	0.001	0.042	[−0.082, 0.084]	*p* = 0.984
Affective numbing	AI work pressure	0.391	0.047	[0.298, 0.483]	*p* < 0.001
	AI autonomy	−0.164	0.043	[−0.249, −0.078]	*p* < 0.001
	Supervisor support	−0.143	0.042	[−0.225, −0.061]	*p* < 0.001
	AI pressure x AI autonomy	0.004	0.051	[−0.096, 0.105]	*p* = 0.932
	AI pressure x supervisor support	0.008	0.051	[−0.091, 0.107]	*p* = 0.873
Sustained work efficiency	AI work pressure	0.246	0.069	[0.110, 0.382]	*p* < 0.001
	Occupational resilience	0.324	0.063	[0.200, 0.448]	*p* < 0.001
	Affective numbing	0.108	0.076	[−0.042, 0.258]	*p* = 0.158
Ethical vigilance	AI work pressure	−0.054	0.060	[−0.173, 0.064]	*p* = 0.370
	Occupational resilience	0.211	0.064	[0.085, 0.337]	*p* = 0.001
	Affective numbing	−0.234	0.066	[−0.364, −0.104]	*p* < 0.001
Professional skepticism	AI work pressure	−0.075	0.059	[−0.190, 0.040]	*p* = 0.202
	Occupational resilience	0.257	0.062	[0.136, 0.378]	*p* < 0.001
	Affective numbing	−0.208	0.066	[−0.337, −0.079]	*p* = 0.002

Standardized predictors are used. HC3 robust standard errors are reported. Because models were estimated using available cases after listwise deletion on variables included in each equation, analytic *N* varied across specifications.

### 4.3 Indirect effects and robustness checks

Table 8 presents prospective indirect effects estimated with Monte Carlo confidence intervals (20,000 draws). The indirect path from AI work pressure through occupational resilience to sustained work efficiency was significant (β = 0.048, SE = 0.017, 95% CI [0.019, 0.085]), indicating that resilience partially mediates the pressure-efficiency relationship. The parallel path through affective numbing to efficiency was not significant (β = 0.043, 95% CI [−0.017, 0.105]), consistent with the null direct effect. The indirect paths from AI pressure through affective numbing to ethical vigilance (β = −0.091, 95% CI [−0.149, −0.039]) and to professional skepticism (β = −0.081, 95% CI [−0.138, −0.029]) were both significant, confirming that affective numbing mediates the negative association between AI pressure and professional judgment outcomes.

**Table 8:** Prospective indirect associations.

Indirect association	Estimate	SE	95% CI	Decision
AI pressure → resilience → efficiency	0.048	0.017	[0.019, 0.085]	Supported
AI pressure → numbing → efficiency (exploratory)	0.043	0.031	[−0.017, 0.105]	Not supported
AI pressure → numbing → ethical vigilance	−0.091	0.028	[−0.149, −0.039]	Supported
AI pressure → numbing → professional skepticism	−0.081	0.028	[−0.138, −0.029]	Supported

Monte Carlo confidence intervals with 20,000 draws.

Table 9 presents robustness checks for the focal affective numbing estimates. Across the base efficiency model (*N* = 298) and the role fixed-effects specification, the numbing-to-efficiency path remained non-significant and virtually identical (β = 0.108 and 0.109, both *p* approximately 0.159), confirming the null finding is not a modeling artifact. In the high AI-use subsample (*n* = 128), the estimate reversed direction (β = −0.102) but remained non-significant (*p* = 0.425), consistent with small-sample imprecision. By contrast, numbing-to-ethical-vigilance (β = −0.234, *p* < 0.001) and numbing-to-professional-skepticism (β = −0.208, *p* = 0.002) were robust across all specifications, reinforcing that affective numbing primarily threatens professional judgment quality rather than task efficiency.

**Table 9:** Robustness checks.

Specification	*N*	Key predictor	β	SE	95% CI	*p*	Interpretation
Base efficiency model	298	Affective numbing	0.108	0.076	[−0.042, 0.258]	*p* = 0.158	Reported cautiously
Role fixed effects	298	Affective numbing	0.109	0.077	[−0.043, 0.260]	*p* = 0.159	Reported cautiously
High AI-use subsample	128	Affective numbing	−0.102	0.128	[−0.352, 0.149]	*p* = 0.425	Reported cautiously
Ethical vigilance outcome	298	Affective numbing	−0.234	0.066	[−0.364, −0.104]	*p* < 0.001	Robust
Professional skepticism outcome	298	Affective numbing	−0.208	0.066	[−0.337, −0.079]	*p* = 0.002	Robust

The focal robustness parameter is affective numbing unless otherwise noted.

In summary, the hypothesis tests supported the proposed dual adaptation pattern but not the moderation predictions. H1 and H2 were supported because AI work pressure was prospectively associated with both occupational resilience and affective numbing. H3 was supported because occupational resilience was prospectively associated with sustained work efficiency. H4a and H4b were supported because affective numbing was associated with lower ethical vigilance and lower professional skepticism. The exploratory efficiency association for affective numbing was not supported, and the hypothesized interaction effects involving AI autonomy and supervisor support were not supported.

### 4.4 Explained variance

Table 10 summarizes the explained variance for the five endogenous variables in the longitudinal path model, complementing the path coefficients reported in Table 7. The T2 adaptation equations explained moderate variance, with R^2^ = 0.28 for occupational resilience and R^2^ = 0.34 for affective numbing. These values indicate that AI work pressure, AI autonomy, supervisor support, interaction terms, and demographic controls collectively accounted for roughly one quarter to one third of individual differences in T2 adaptation states. The affective numbing equation explained more variance than the resilience equation, consistent with the stronger direct effect of AI pressure on numbing than on resilience (β = 0.391 vs. β = 0.149). The T3 outcome equations showed lower explained variance, with R^2^ = 0.18 for sustained work efficiency, R^2^ = 0.21 for ethical vigilance, and R^2^ = 0.19 for professional skepticism. This pattern is expected given the twelve week prospective lag and the modest attrition related restriction of range noted earlier.

**Table 10:** Explained variance (R^2^) for endogenous variables in the longitudinal path model.

Outcome variable	Wave	R^2^	Predictors included
Occupational resilience	T2	0.28	AI work pressure, AI autonomy, supervisor support, interaction terms, controls
Affective numbing	T2	0.34	AI work pressure, AI autonomy, supervisor support, interaction terms, controls
Sustained work efficiency	T3	0.18	Occupational resilience, affective numbing, AI work pressure, controls
Ethical vigilance	T3	0.21	Occupational resilience, affective numbing, AI work pressure, controls
Professional skepticism	T3	0.19	Occupational resilience, affective numbing, AI work pressure, controls

R^2^ values are from the full longitudinal path model estimated in R/lavaan. Controls include age, gender, tenure, weekly work hours, and baseline well-being.

## 5 Discussion

This study introduced and tested the numb efficiency paradox in a longitudinal sample of accounting and auditing professionals who use AI enabled work tools. The core pattern was broadly supported. AI work pressure was associated with both occupational resilience and affective numbing. Occupational resilience was prospectively associated with later sustained work efficiency, whereas affective numbing was not. Affective numbing, however, was associated with lower ethical vigilance and professional skepticism. These findings indicate that sustained efficiency under AI pressure is not a psychologically homogeneous outcome. Similar levels of task output may be maintained by employees who remain professionally engaged and by employees who have become affectively detached from the warning signals on which professional judgment depends. The distinction matters because efficiency metrics can show that work is being completed, but they cannot reveal whether the psychological conditions supporting careful judgment, ethical attention, and professional doubt are being preserved.

This distinction has particular theoretical importance in accounting and auditing because professional judgment is not merely a desirable work behavior, but a core feature of regulated professional practice. Auditing and accounting operate within explicit quality standards, including International Standards on Auditing, Generally Accepted Auditing Standards, and regulatory oversight by bodies such as the PCAOB and FRC. Within this framework, questioning AI generated estimates, flagging unusual journal entries, and resisting pressure to accept automated outputs without independent verification are not simply individual preferences. They are part of the professional responsibility through which audit and accounting quality is maintained. Affective numbing may be linked with lower emotional responsiveness to the cues that make such judgment moments salient. An auditor who continues to complete engagement tasks efficiently but reacts less strongly to exception alerts, inconsistent reconciliations, or aggressive estimates may satisfy throughput metrics while becoming less attentive to the judgment standards that the work requires. Conservation of resources theory suggests that sustained resource pressure may be associated with states in which employees remain surface functional while becoming internally depleted or defensive (Hobfoll, 1989). The present findings extend this logic by showing that affective numbing, understood as reduced emotional responsiveness to occupational signals, is linked less clearly to efficiency loss than to diminished professional sensitivity. The central theoretical distinction is therefore not between working and not working, but between resilient engagement and detached persistence. Resilience implies recovery, flexibility, and preserved professional attention, whereas affective numbing reflects reduced responsiveness to repeated pressure and professional warning cues. This makes numbing distinct from burnout, disengagement, moral disengagement, and automation bias, because it concerns the affective and evaluative sensitivity with which employees approach AI assisted professional judgment.

### 5.1 Theoretical contributions

The study contributes to AI workplace research by showing that AI work pressure may be linked to both adaptive and maladaptive forms of persistence, thereby moving technostress research beyond a simple performance versus strain framing. By distinguishing resilient efficiency from detached persistence, the findings refine performance centered views of AI implementation and suggest that AI related productivity gains in professional service contexts should be evaluated alongside the psychological conditions through which such productivity is maintained. The contribution is especially relevant to accounting and auditing because the study identifies affective numbing as a psychological pathway linking AI mediated pressure with lower professional judgment sensitivity without necessarily corresponding to lower efficiency based performance metrics. This finding speaks to longstanding concerns in audit quality research regarding the conditions under which professional skepticism is weakened, the role of client pressure and cognitive load in reducing auditor objectivity, and the behavioral consequences of decision aid reliance (Nelson, 2009; Bamber and Iyer, 2007; Dowling and Leech, 2014). The nonsignificant interaction effects further suggest that autonomy and supervisor support may not automatically buffer the association between AI pressure and numbing within the time window examined here. Instead, these resources appeared to operate mainly as general resources, given their direct associations with higher resilience and lower affective numbing. This pattern is consistent with conservation of resources theory, which suggests that resources may show additive benefits under sustained demand rather than always moderating the slope of stressor associations (Hobfoll, 1989). It is also possible that the six week interval between T1 and T2 was too short to detect cumulative buffering processes. Longer panel studies with more frequent measurement occasions could examine whether buffering effects emerge over time, and future research could also investigate resource caravan effects, including whether multiple coexisting resources jointly protect professional sensitivity in AI intensive accounting environments (Hobfoll et al., 2018).

### 5.2 Practical implications

Practically, accounting firms should not evaluate AI implementation only through speed, throughput, or short term efficiency. Organizations also need to monitor whether employees remain ethically attentive and professionally skeptical when working with AI supported workflows. The present findings suggest several practical directions. AI workflow design should include structured judgment checkpoints at which employees document the reasoning behind their evaluation of AI outputs, not merely whether they accepted or rejected an automated recommendation. Such checkpoints can encourage active professional evaluation, reduce passive acceptance, and provide an audit trail for later review. Engagement quality reviews may also incorporate behavioral indicators of professional engagement, including the frequency of auditor initiated inquiries, the rate of AI output overrides, and the quality of documented skeptical reasoning, alongside conventional efficiency metrics. Supervisor support should further legitimize doubt, caution, and the expression of ethical discomfort, rather than implicitly rewarding only speed and output volume. In AI intensive audit environments, a supervisor who validates an associate’s decision to challenge an automated journal entry flag may help counteract the detached passivity associated with affective numbing. These recommendations are consistent with guidance emphasizing that technology tools should support rather than replace professional judgment in audit processes. They also align with audit judgment research showing that information abundance and automated decision aids create behavioral demands that cannot be managed through efficiency optimization alone (Brown-Liburd et al., 2015; DeFond and Zhang, 2014; Dowling and Leech, 2014).

### 5.3 Limitations and future research

Several limitations should be acknowledged alongside directions for future research. First, the study relied on self reported measures for all focal constructs, which introduces the possibility of common method variance even though the three wave design reduced same time measurement bias. This limitation is particularly important for ethical vigilance and professional skepticism, because participants’ self evaluations may not fully capture the behavioral skepticism that appears in observable audit actions, such as inquiry frequency, workpaper documentation quality, fraud risk questioning, and the willingness to override or challenge AI generated outputs. Future research should therefore supplement self report measures with objective or semi objective indicators of professional judgment. Useful designs would include audit simulation tasks, fraud detection scenarios, objective error detection performance, supervisor rated audit quality assessments, case based judgment tasks adapted from audit research, behavioral audit outcomes, and AI use log data capturing override rates, inquiry behavior, and time on task in real audit software environments. Such behavioral and archival indicators would allow researchers to test whether affective numbing is associated not only with perceived reductions in ethical vigilance, but also with measurable changes in the judgment behaviors required by professional standards. Second, the professional panel sample limits generalizability. Although the Prolific-based sample provided occupational specificity through prescreening, qualification checks, and study-specific eligibility screeners, it was restricted to four English-speaking countries and may not represent global accounting professionals, non-English-speaking contexts, public-sector accounting environments, or organizations with substantially different levels of AI adoption and governance. Replication with organizationally embedded samples, including Big 4 and non Big 4 audit firms, internal audit teams, shared service centers, and non-Anglophone jurisdictions, would strengthen external validity. Third, attrition analyses indicated that employees experiencing greater AI pressure were somewhat less likely to complete all waves. This pattern means that the final longitudinal sample may underrepresent the professionals most strongly affected by AI mediated pressure. As a result, the reported effect sizes may be conservative, particularly for the associations involving AI pressure and affective numbing. Future studies should use retention-enhancing designs, planned missing-data procedures, and sensitivity analyses to evaluate how selective attrition shapes estimates.

A fourth limitation concerns the measurement and developmental timing of affective numbing. Affective numbing was newly operationalized in an occupational context, and although the discriminant validity evidence indicated separation from emotional exhaustion, depersonalization, and automation bias, these tests were conducted within the same sample as the focal analyses. The scale should therefore be treated as a theoretically grounded but still developing measure rather than as a fully established instrument. Future validation research should examine affective numbing alongside well validated burnout measures, test its incremental prediction of professional judgment outcomes, assess convergent validity with adapted measures of emotional numbing from trauma and compassion fatigue research, report independent EFA and CFA evidence, and evaluate test retest reliability across shorter intervals. The six week and twelve week intervals between waves may also be insufficient to capture the full development of numbing under sustained AI pressure. Experience sampling and diary designs would be better suited to tracing how pressure, emotional responsiveness, and professional judgment fluctuate within real accounting workflows. The unsupported moderation hypotheses should also be interpreted cautiously. The power analysis indicated adequate power to detect moderate interaction effects, which suggests that the nonsignificant interaction terms are unlikely to be explained solely by insufficient sample size. However, the absence of moderation may also reflect the time window, the measurement of contextual resources, or the possibility that AI autonomy and supervisor support operate mainly as general resources rather than as buffers of the pressure to numbing relationship. Longer panel designs with more frequent measurement occasions could clarify whether buffering effects emerge cumulatively over time. Future applied research should also examine whether organizational AI governance, explainable AI interfaces, formal human oversight protocols, and structured escalation mechanisms reduce affective numbing by making professional accountability clearer and by legitimizing skeptical challenge of automated outputs.

## 6 Conclusion

AI enabled accounting work raises a new psychological question: when employees remain efficient under sustained pressure, are they resilient, or have they become affectively numb? This study provides longitudinal evidence that the distinction matters and that efficiency metrics alone cannot answer it. Occupational resilience and affective numbing were both prospectively associated with AI work pressure, but they diverged in their associations with later outcomes. Resilience was linked to sustained efficiency and preserved professional engagement, whereas affective numbing was not a reliable source of efficiency and was associated with lower ethical vigilance and professional skepticism. The numb efficiency paradox captures this pattern: efficient appearing behavior may coexist with reduced professional sensitivity. This paradox points to a structural blind spot in AI enabled accounting work, because workflow metrics, completion rates, and deadline adherence reveal whether work is being completed, but not whether the psychological conditions for careful professional judgment are being preserved. For accounting practice, the implication is clear. AI implementation should be evaluated not only by speed and productivity, but also by whether employees remain attentive to anomalies, ethically responsive to warning signs, and willing to question automated outputs. Auditing standards across major jurisdictions require professional skepticism throughout the engagement, yet conventional performance systems may not detect affective numbing when task output remains stable. The present findings therefore suggest the need to complement procedural quality controls with behavioral indicators of professional engagement. Practical steps include incorporating indicators such as auditor initiated inquiries, AI output challenge rates, and documentation of skeptical reasoning into performance and quality review systems; training supervisors to recognize and respond to signs of detached persistence; and designing AI assisted workflows that require explicit human judgment at decision critical points rather than passive review of automated outputs. Future research, practitioners, and standard setters should work together to ensure that AI enabled accounting delivers efficiency gains without weakening the professional judgment on which the legitimacy of accounting and auditing depends.

## Data Availability

The original contributions presented in the study are included in the article/Supplementary material, further inquiries can be directed to the corresponding author.
