# Supplementary material for: The numb efficiency paradox: AI work pressure, affective numbing, and professional judgment in organizationally digitally mediated work
Source: Front Psychol. 2026 Aug 28;17:1900940. doi: 10.3389/fpsyg.2026.1900940 (PMC13561801; doi:10.3389/fpsyg.2026.1900940)
Supplement: Supplementary file 1 [file Table_1.docx]

Appendix A continued. Exploratory Factor Evidence

Note. Eigenvalues were computed from Pearson item correlation matrices. Extraction used principal-axis factoring. Construct-level evidence is based on one-factor extraction for each construct; overall evidence is based on a nine-factor principal-axis extraction with varimax rotation across all focal items.

Table A1. Construct-level eigenvalues, minimum factor loading, and extraction summary

| **Construct** | **N** | **Items** | **1st eigenvalue** | **2nd eigenvalue** | **Min loading** | **Mean extraction** |
| --- | --- | --- | --- | --- | --- | --- |
| AI-mediated work pressure | 512 | 6 | 2.871 | 0.750 | 0.519 | 0.376 |
| AI autonomy | 512 | 4 | 2.261 | 0.651 | 0.598 | 0.422 |
| Supervisor support | 512 | 4 | 2.530 | 0.540 | 0.702 | 0.510 |
| Baseline well-being | 512 | 4 | 2.366 | 0.603 | 0.628 | 0.456 |
| Occupational resilience | 417 | 5 | 2.844 | 0.630 | 0.637 | 0.462 |
| Affective numbing | 417 | 6 | 3.222 | 0.664 | 0.574 | 0.446 |
| Sustained work efficiency | 361 | 4 | 2.344 | 0.612 | 0.612 | 0.450 |
| Ethical vigilance | 361 | 5 | 2.811 | 0.614 | 0.620 | 0.454 |
| Professional skepticism | 361 | 4 | 2.332 | 0.676 | 0.549 | 0.449 |

Table A2. Item-level factor loadings and extraction communalities

| Construct | No. of items | Loading range | Communality range |
| --- | --- | --- | --- |
| AI-mediated work pressure | 6 | 0.519–0.673 | 0.269–0.453 |
| AI autonomy | 4 | 0.598–0.710 | 0.358–0.505 |
| Supervisor support | 4 | 0.702–0.737 | 0.493–0.544 |
| Baseline well-being | 4 | 0.628–0.701 | 0.394–0.491 |
| Occupational resilience | 5 | 0.637–0.717 | 0.406–0.515 |
| Affective numbing | 6 | 0.574–0.740 | 0.329–0.547 |
| Sustained work efficiency | 4 | 0.612–0.725 | 0.375–0.526 |
| Ethical vigilance | 5 | 0.620–0.734 | 0.384–0.539 |
| Professional skepticism | 4 | 0.549–0.744 | 0.301–0.553 |

Note. Principal-axis factoring was used. Across constructs, factor loadings ranged from 0.519 to 0.744. All retained items loaded above 0.50 on their intended construct.

Table A3. Overall item-level eigenvalues

| **Factor** | **Eigenvalue** | **% variance** | **Cumulative %** | **Eigenvalue > 1** |
| --- | --- | --- | --- | --- |
| 1 | 7.292 | 17.363 | 17.363 | True |
| 2 | 4.420 | 10.525 | 27.887 | True |
| 3 | 2.800 | 6.666 | 34.553 | True |
| 4 | 2.036 | 4.846 | 39.399 | True |
| 5 | 1.881 | 4.479 | 43.879 | True |
| 6 | 1.697 | 4.041 | 47.919 | True |
| 7 | 1.611 | 3.835 | 51.754 | True |
| 8 | 1.385 | 3.298 | 55.052 | True |
| 9 | 1.114 | 2.652 | 57.705 | True |
| 10 | 0.899 | 2.140 | 59.845 | False |
| 11 | 0.876 | 2.085 | 61.930 | False |
| 12 | 0.847 | 2.016 | 63.946 | False |
